# *Educational Resource Review:* MSD’s The Steward—Episode 2—Drug-resistant infections: the patient perspective

**DOI:** 10.1093/jacamr/dlab059

**Published:** 2021-05-24

**Figure:**
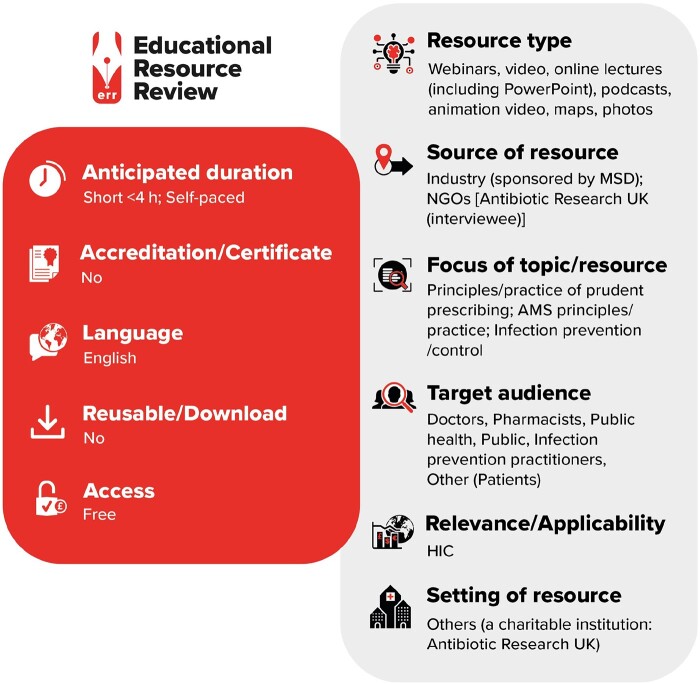


LI, low-income countries; LMIC, low- and middle-income countries; HMI, high- and middle-income countries; HIC, high-income countries.

**Resource web link:** https://www.youtube.com/watch?v=KeUTD7w4Zxg (Full classification scheme available at: http://bsac.org.uk/wp-content/uploads/2019/03/Educational-resource-review-classification-scheme.pdf)

**WHO region and country (World Bank):** European Region, UK (HIC)

## Peer review commentary

This is the second episode of a series of MSD-sponsored interviews entitled ‘The Steward’. If this episode is anything to go by, the other episodes are also very interesting. This episode is an eye opener for anyone involved in antimicrobial resistance/antimicrobial stewardship (AMR/AMS) on how to translate these terms to the here and now, using terminology that a patient can understand and avoiding terms such as ESBL, MDR, etc. However, it might be also relevant to tone down the microbiological jargon when conveying a message to clinicians and other healthcare professionals who might not be infection experts. This makes the interview applicable to anyone from the medical field through patients’ groups, patients and relatives. 

The interviewee takes an important, bold stance that speaking about ‘10 million deaths by 2050’ or that it will cost ‘100 trillion dollars in excess healthcare costs’ (‘Antimicrobial resistance: tackling a crisis for the health and wealth of nations’) puts the emphasis on ‘the next 3 decades’ or so rather than the present (i.e. not urgent). The only statements subject to misinterpretation were when a ‘Greta Thunberg for AMR’ was mentioned, as they also referred to ‘natural products’. Unfortunately, this could be misinterpreted as stating that natural remedies (or homeopathic treatments) are suitable to treat infections. Another issue is the reference to a patient on life-long rotation of antibiotic cocktails following a surgical procedure. It is understood that patients with, say, cystic fibrosis might benefit from cycling antibiotic prophylaxis, but to advocate this with the general public or even non-infection expert healthcare workers might be subject to misinterpretation and suggest that antibiotics can be used on a long-term basis in general. 

The patient (or patient support group) perspective on AMR/AMS is often lacking, thus making this interview original and applicable across the various aspects of healthcare from providers to recipients. 

